# Limonoid Triterpene, Obacunone Increases Runt-Related Transcription Factor 2 to Promote Osteoblast Differentiation and Function

**DOI:** 10.3390/ijms22052483

**Published:** 2021-03-02

**Authors:** Kyung-Ran Park, SooHyun Kim, MyoungLae Cho, Hyung-Mun Yun

**Affiliations:** 1 Department of Oral and Maxillofacial Pathology, School of Dentistry, Kyung Hee University, Seoul 02447, Korea; rudfks282@naver.com; 2 National Institute for Korean Medicine Development, Gyeongsan 38540, Korea; beluga81@nikom.or.kr (S.K.); meanglae@nikom.or.kr (M.C.)

**Keywords:** *D. dasycarpus*, obacunone, osteoblast differentiation, RUNX2

## Abstract

Root bark of *Dictamnus dasycarpus* Turcz. has been widely used as a traditional medicine and is a well-known anti-inflammatory agent. We isolated limonoid triterpene, obacunone (Obac) from the dried root bark of *D. dasycarpus*. Obac has been reported to exhibit varieties of biological activities including anti-inflammatory, anti-cancer, and anti-oxidant effects. This study aimed to investigate the beneficial effects and biological mechanisms of Obac in osteoblast differentiation and bone matrix mineralization. In the present study, Obac at concentrations ranging from 1 to 100 μM showed no proliferation effects in MC3T3-E1. The treatment of Obac (1 and 10 μM) increased wound healing and migration rates in a dose-dependent manner. Alkaline phosphatase (ALP) staining and activity showed that Obac (1 and 10 μM) enhanced early osteoblast differentiation in a dose-dependent manner. Obac also increased late osteoblast differentiation in a dose-dependent manner, as indicated by the mineralized nodule formation of ARS staining. The effects of Obac on osteoblast differentiation was validated by the levels of mRNAs encoding the bone differentiation markers, including *Alp*, bone sialoprotein (*Bsp*), osteopontin (*Opn*), and osteocalcin (*Ocn*). Obac increased the expression of bone morphogenetic protein (BMP), and the phosphorylation of smad1/5/8, and the expression of runt-related transcription factor 2 (RUNX2); Obac also inhibited GSK3β and upregulated the protein level of β-catenin in a dose-dependent manner during osteoblast differentiation. Obac-mediated osteoblast differentiation was attenuated by a BMP2 inhibitor, Noggin and a Wnt/β-catenin inhibitor, Dickkopf-1 (Dkk1) with the abolishment of RUNX2 expression and nuclear accumulation by Obac. Taken together, the findings of this study demonstrate that Obac has pharmacological and biological activates to promote osteoblast differentiation and bone mineralization through BMP2, β-catenin, and RUNX2 pathways, and suggest that Obac might be a therapeutic effect for the treatment and prevention of bone diseases such as osteoporosis and periodontitis.

## 1. Introduction

The root bark of *Dictamnus dasycarpus* Turcz. (Dictamni Radicis Cortex) is a herb clinically used to treat inflammatory skin diseases such as contact dermatitis, pruritus vuluae, eczema, and scabies [1,2]. Known constituents of *D. dasycarpus* root bark include limonoid triterpene, obacunone (Obac) [3]. It was reported that Obac synergistically increases the cytotoxicity of vincristine against L1210 cancer cells and multidrug-resistant KB-V1 cancer cells, and also inhibits SW480 cancer cell proliferation [4,5]. Obac showed significant neuroprotective activity against glutamate toxicity in cortical and hippocampal neurons [6,7]. Obac has been shown to have nuclear factor erythroid 2-related factor 2-dependent antioxidant activities and suppressed pro-inflammatory mediators through macrophage migration inhibitory factors [8,9]. However, its biological activities have not been demonstrated yet in osteoblasts.

Osteoblast differentiation and function are tightly regulated by physiological and dynamic processes for bone development, formation, and remodeling through the synthesis of bone specific proteins and the mineralization of the organic bone matrix throughout life [10]. Osteoblasts also actively participate in bone repair processes, including the migration, differentiation, and bone matrix mineralization after bone injury and fracture [11,12]. The impairment and dysfunction of osteoblasts are closely involved in the pathogenesis of bone diseases such as osteoporosis and periodontitis [10,13,14,15]. Therefore, osteoblasts are an important target to treat bone diseases using pharmacological approaches [10,16,17,18]. However, it is much more difficult to treat bone diseases, because drugs such as parathyroid hormone, bisphosphonates, and calcitonin have serious adverse effects with safety and efficiency issues [13,17,19,20].

Natural compounds have been identified and developed as potential drugs to treat bone diseases because they are relatively inexpensive and safe compared with chemically synthesized drugs, and have also been used as traditional medicine for centuries [10,18]. Thus, it is essential to study potential compounds from plants based on biological mechanisms to translate the knowledge into bone disease treatment.

In the present study, we isolated Obac (purity 99.8%) from the root bark of *D. dasycarpus*, and we investigated the underlying mechanism and biological effects of Obac on cell viability, migration, osteoblast differentiation, and matrix mineralization in MC3T3-E1.

## 2. Results

### 2.1. Obac Isolated from D. dasycarpus Dried Root Bark Has No Cytotoxic and Proliferative Effects in MC3T3-E1

**Figure 1:**
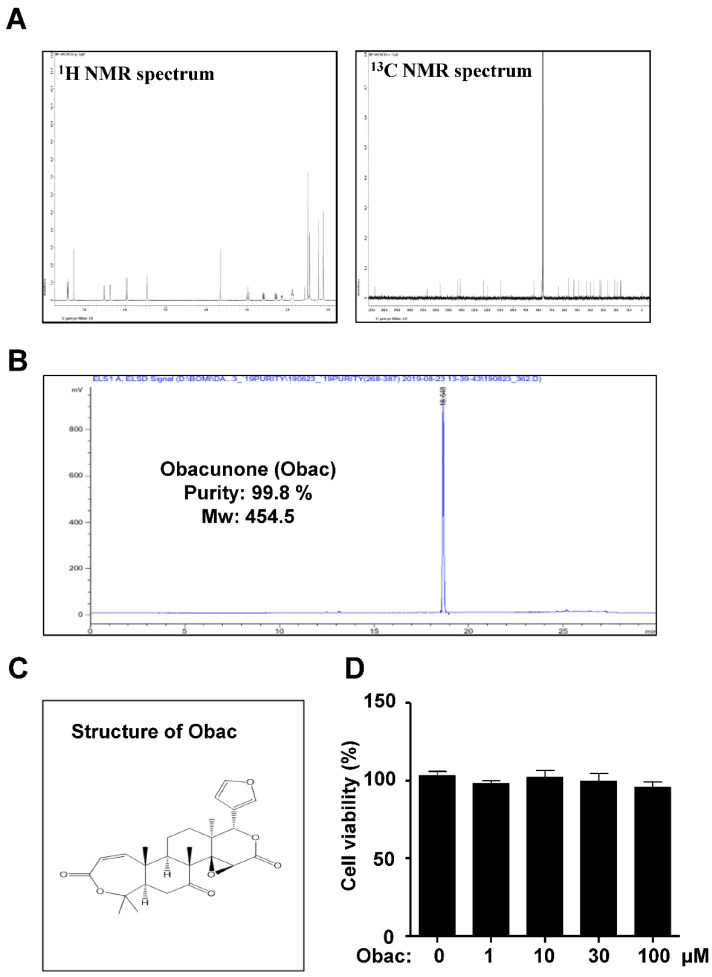
Effects of Obac on proliferation in MC3T3-E1. (**A**) ^1^H and ^13^C NMR spectra of Obac from the dried root bark of *Dictamnus dasycarpus* Turcz. (**B**) HPLC chromatogram of Obac. (**C**) Chemical structure of Obac. (**D**) MC3T3-E1 was incubated with Obac at concentrations of 1, 10, 30, and 100 μM for 24 h, and cell viability was measured by the MTT assay. Data are representative of three independent experiments, and values are expressed in the mean ± S.E.M

Obac (99.8% purity) was isolated from the dried root bark of *D. dasycarpus* limonoid triterpene, and the structure of Obac was determined by comparison of its spectroscopic data with that reported in previous literature [5] (Figure 1A–C). Firstly, in order to investigate the biological effects of Obac on the proliferation in MC3T3-E1, Obac (1, 10, 30, and 100 μM) were treated for 24 h, and cell viability (%) was determined using an MTT assay and BrdU incorporation assay. In MC3T3-E1, Obac did not affect proliferation at concentrations ranging from 1 to 100 μM (Figure 1D, Supplementary Materials Figure S1).

### 2.2. Obac Enhances Cell Migration in Early Osteoblast Differentiation

**Figure 2:**
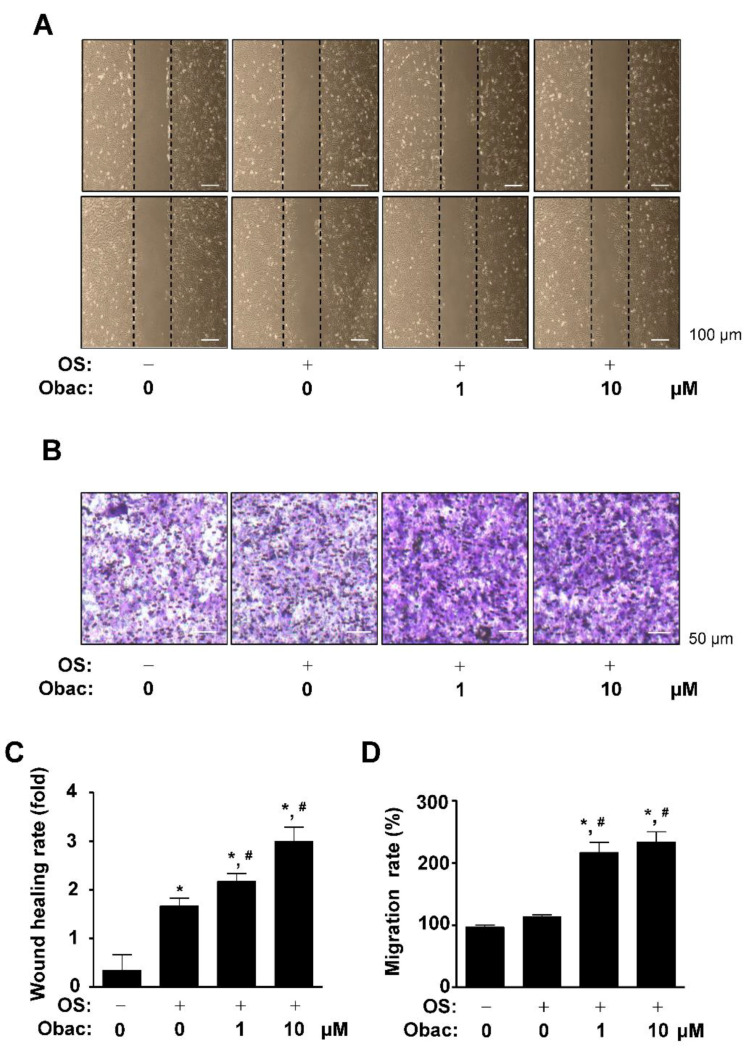
Effects of Obac on cell migration in osteoblast differentiation. (**A**,**C**) MC3T3-E1 was cultured in osteogenic supplement medium (OS) in the absence or presence of Obac (1–10 µM) for 24 h. Cell migration was observed under a phase contrast microscope (**A**), and the cell migration rate (fold) was measured by the area enclosing the spreading cell population and expressed as a bar graph (**C**). (**B**,**D**) The Boyden chamber assay was performed and observed under a phase contrast microscope (**B**), and migration rate (%) was normalized to the control and expressed as a bar graph (**D**). Data are representative of three independent experiments, and values are expressed as the mean ± S.E.M. * *p* < 0.05 indicates statistically significant differences, compared with the control. #: statistically significant differences compared with OS (^#^ *p* < 0.05).

The migration of mesenchymal stem cells is upregulated in early osteoblast differentiation, and migration is decreased but adhesiveness is increased at a later stage [21]. The migration of pre-osteoblasts is also critical for the process of osteogenesis in bone formation and bone tissue repair [22,23]. Thus, we next examined whether Obac has biological effects on cell migration in osteoblast differentiation. For wound healing assay, the area of the wound was measured immediately after scratching, and osteoblast differentiation was induced using osteogenic supplement medium (OS) in Obac (0–10 μM) for 24 h. Cells moved forward over the wound area, and wound healing rate was significantly increased in a dose-dependent manner (Figure 2A,C). To further validate Obac-mediated cell migration in osteoblast differentiation, effects of Obac were examined in Boyden chamber assays. Obac treatment showed that cell transmigration was significantly enhanced in a dose-dependent manner (Figure 2B,D).

### 2.3. Obac Enhances ALP-Positive Cells and Mineralized Nodule Formation in Early and Late Osteoblast Differentiation

**Figure 3:**
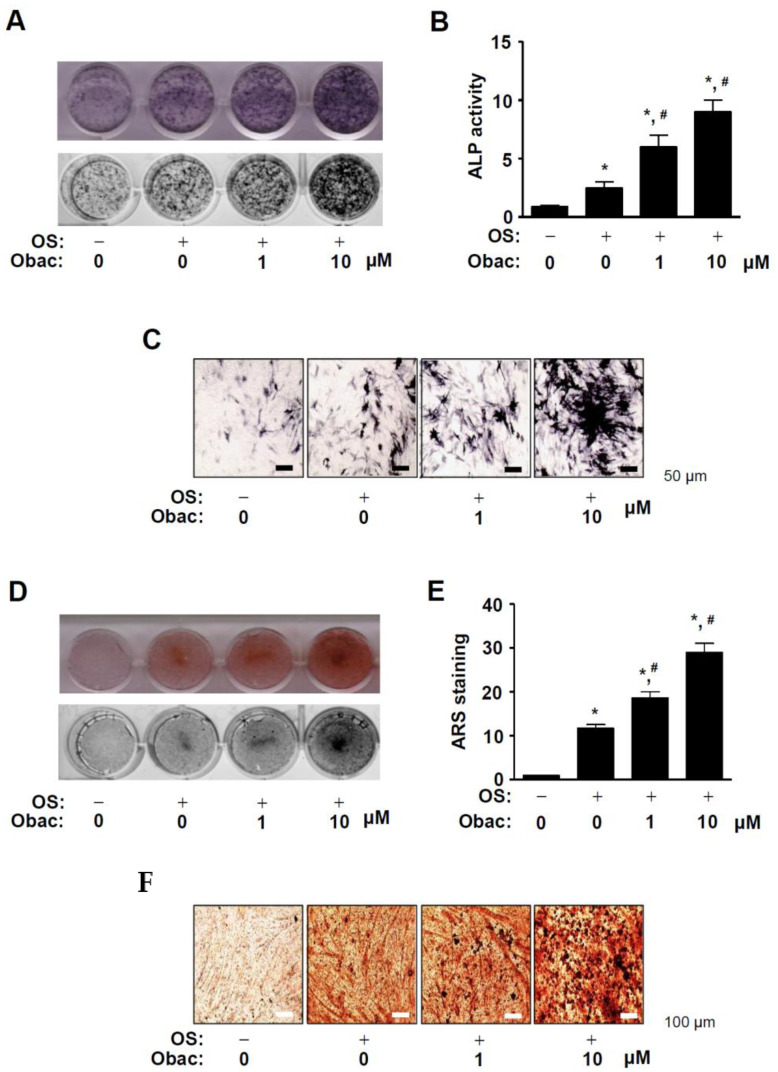
Effects of Obac on early and late osteoblast differentiation. (**A**–**C**) MC3T3-E1 was incubated in OS with Obac (1–10 µM) for 7 days. ALP staining was detected using a scanner (upper) and a colorimetric detector (bottom) (**A**), the enzymatic activity of alkaline phosphatase (ALP) was measured at 405 nm using a spectrophotometer (**B**), and ALP-positive cells were observed using a light microscope (**B**). Scale bar: 50 μm. (**D**–**F**) After osteoblast differentiation for 14 days, the mineralization formation was analyzed using Alizarin red S (ARS) staining. The ARS staining was detected using a scanner (upper) and a colorimetric detector (bottom) (**D**), quantified by measuring at a wavelength of 590 nm using a spectrophotometer (**E**), and the mineralized nodules were observed using a light microscope (**F**). Scale bar: 100 μm. Data are representative of three independent experiments, and values are expressed as the mean ± S.E.M. * *p* < 0.05 indicates statistically significant differences, compared with the control. #: statistically significant difference compared with OS (^#^ *p* < 0.05).

To demonstrate the biological activities of Obac on early osteoblast differentiation, we induced osteoblast differentiation using OS with Obac (0–10 μM) for 7 days, and the early osteoblast differentiation was observed by staining alkaline phosphatase (ALP) using a digital camera and a colorimetric detector. ALP stain and activity were increased in response to Obac treatment in a dose-dependent manner (Figure 3A,B). ALP-positive-stained cells in response to Obac treatment were also observed using a light microscope (Figure 3C).

To further investigate the biological activities of Obac on late osteoblast differentiation, we measured matrix mineralization using Alizarin red S (ARS) staining after osteoblast differentiation was induced with Obac (0–10 μM) for 14 days. As shown in Figure 3D,E, Obac treatment increased mineralized nodule formation in a dose-dependent manner. Consistent with the observations, Obac-mediated mineralization was also observed on a light microscope (Figure 3F).

### 2.4. Obac Upregulates Osteoblast Gene Expression in Osteoblast Differentiation

**Figure 4:**
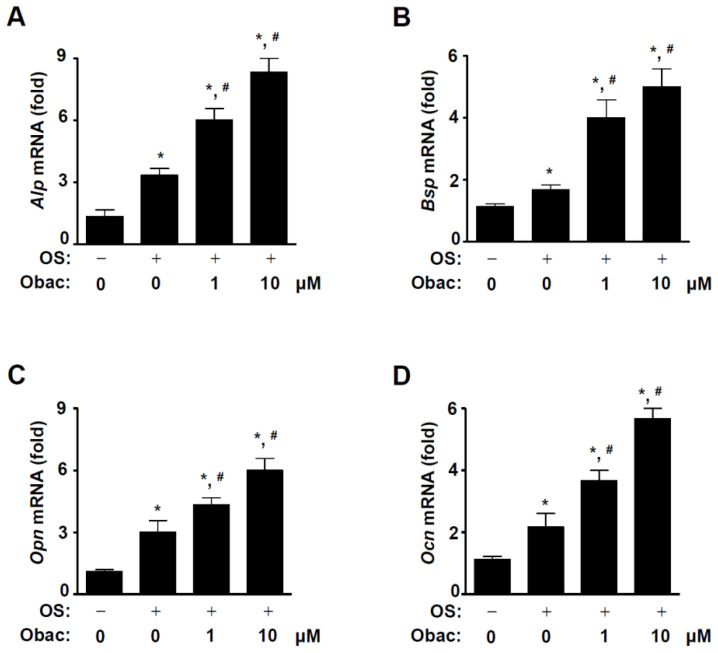
Effect of Obac on the expression of mRNAs encoding the bone differentiation markers in osteoblast differentiation. (**A**–**D**) MC3T3-E1 was incubated with Obac (1–10 µM) for 7 days in osteoblast differentiation, and the total RNA was isolated, osteoblast-marker genes including *Alp* (**A**), *Bsp* (**B**), *Opn* (**C**), *Ocn* (**D**) were analyzed, and then the target gene levels were normalized to β-actin. Data are representative of three independent experiments, and values are expressed in the mean ± S.E.M. * *p* < 0.05 indicates statistically significant difference, compared with the control. #: statistically significant difference compared with OS (^#^ *p* < 0.05).

Next, we investigated the biological effects of Obac on mRNA expression for osteoblast differentiation. MC3T3-E1 was differentiated for 7 days in Obac (0–10 μM). As shown in Figure 4A–D, Obac enhanced levels of mRNAs encoding the bone differentiation markers including Alp, bone sialoprotein (*Bsp*), osteopontin (*Opn*), and osteocalcin (*Ocn*), which is the target genes of RUNX2, a key transcription factor that plays a prominent role in osteoblast differentiation.

### 2.5. Obac Activates BMP2-Smad1/5/8 and β-Catenin Pathways, and Increases RUNX2 Expression in Osteoblast Differentiation

**Figure 5:**
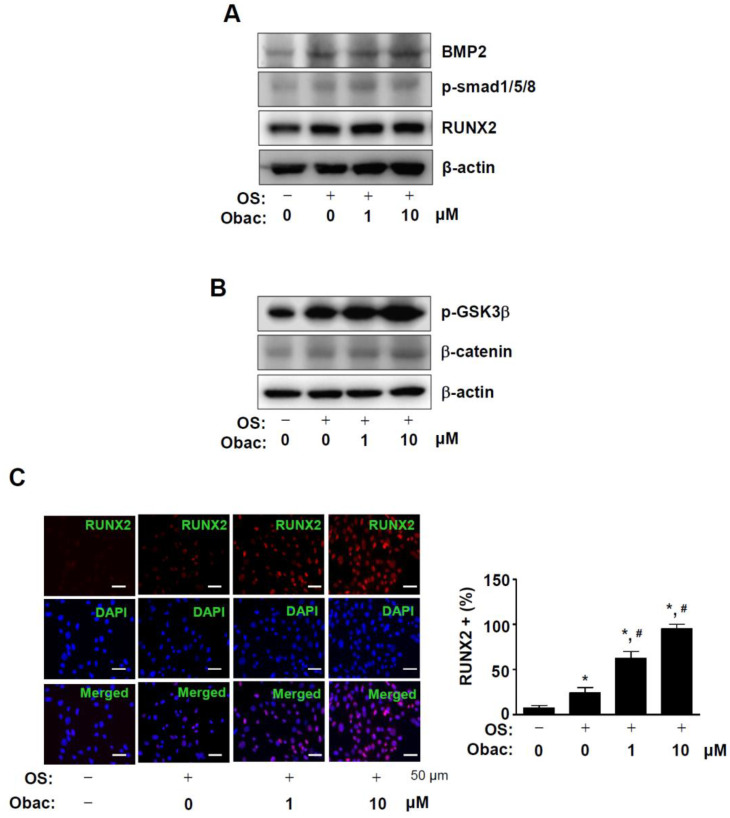
Effect of Obac on BMP2 and β-catenin signaling, and RUNX2 expression in osteoblast differentiation. (**A**,**B**) After 48 h, the equal amounts of lysates were assessed using antibodies against BMP2, phospho-Smad1/5/8 (p-Smad1/5/8), and RUNX2 (**A**), and phospho-GSK3β (p-GSK3β) and β-catenin (**B**). β-actin was detected on the same sample to normalize the values obtained using Western blot analysis. (**C**) RUNX2 was immunostained with rabbit anti-RUNX2 antibody, followed by Alexa-Fluor 568-conjugated secondary antibody (red). After the cells were counterstained with DAPI (blue), the slides were mounted and visualized by a fluorescence microscope. The third panel shows the merged images of the first and second panels. RUNX2+ cells (%) were expressed in a bar graph. Scale bar: 50 μm. Data are representative of three independent experiments.

To determine the biological mechanism of Obac for osteoblast differentiation, bone morphogenetic protein (BMP) and β-catenin pathways were examined in MC3T3-E1. The treatment of Obac (1 and 10 μM) significantly increased the intracellular expression of BMP2 and the phosphorylation of Smad1/5/8, and also upregulated the expression of RUNX2 (Figure 5A). As shown in Figure 5B, the treatment of Obac (1 and 10 μM) increased the phosphorylation of GSK3β and the protein level of β-catenin in osteoblast differentiation. We further confirmed the expression of RUNX2 in the nucleus using immunofluorescence assays. These results revealed that the treatment of Obac (1 and 10 μM) increased the nuclear accumulation of RUNX2 in osteoblast differentiation (Figure 5C, Supplementary Figure S2).

### 2.6. Obac Causes Osteoblast Differentiation by RUNX2 Expression through the BMP2 and β-Catenin Pathways

**Figure 6:**
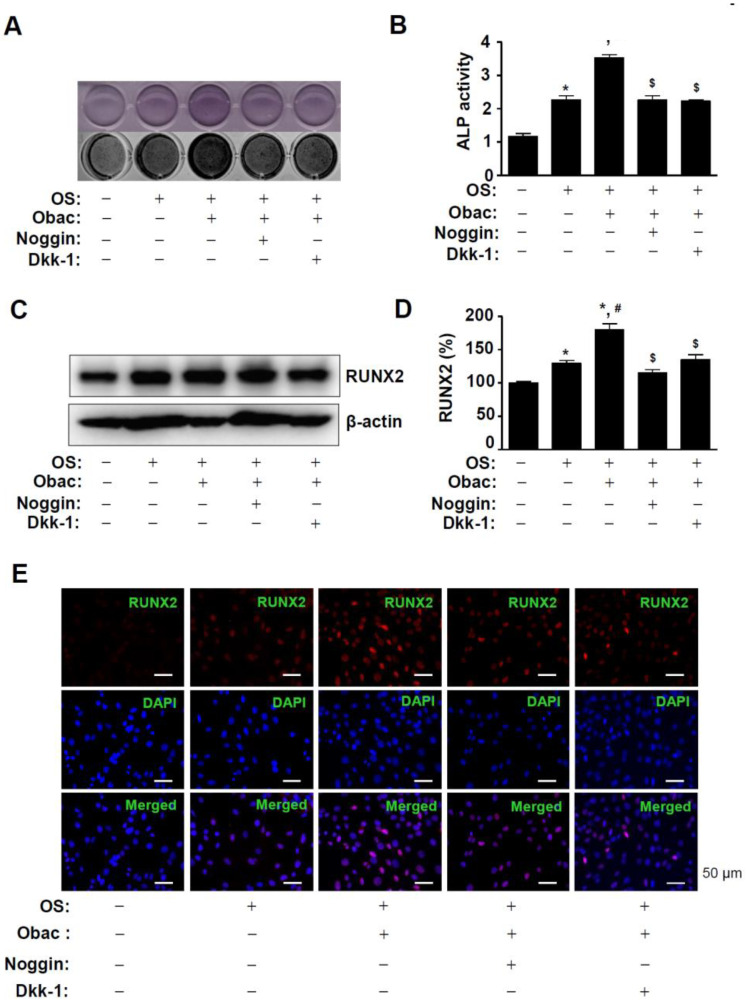
Effects of Obac-induced osteoblast differentiation and RUNX2 expression through the inhibition of BMP2 and β-catenin signaling. (**A**,**B**) MC3T3-E1 was incubated in OS with 10 µM Obac in the absence or presence of noggin (10 µg/mL) and Dkk-1 (0.5 µg/mL) for 7 days. ALP staining was detected using a scanner (upper) and a colorimetric detector (bottom) (**A**), and ALP activity was assessed using ALP activity colorimetric assay (**C**). (**C**,**D**) After 48 h, the expression of RUNX2 was assessed using Western blot analysis (**C**). β-actin was detected on the same sample to normalize the values, and the data were expressed as a bar graph (**D**). (**E**) After 48 h, the nuclear localization of RUNX2 were assessed using Immunofluorescence. The first panels show RUNX2 expression (red), the second panels show DAPI (a nuclear marker, blue), and the bottom panels show the merged images of the first and second panels. Scale bar: 50 μm. Data are representative of three independent experiments, and values are expressed in the mean ± S.E.M. * *p* < 0.05 indicates statistically significant differences, compared with the control. #: statistically significant differences compared with OS (^#^ *p* < 0.05). $: statistically significant differences compared with the OS + Obac (^$^ *p* < 0.05).

In order to examine whether Obac induced osteoblast differentiation through the BMP2 and β-catenin pathways, Obac was treated in the absence and presence of a Wnt/β-catenin inhibitor, Dickkopf-1 (Dkk-1) and a BMP inhibitor, noggin. The pretreatment of cells with Dkk-1 and noggin significantly attenuated Obac-stimulated ALP staining and ALP enzymatic activity in osteoblast differentiation (Figure 6A,B). To demonstrate the functional link the between the BMP2 and β-catenin pathways, we examined the effects of Obac on RUNX2. Dkk-1 and noggin abolished the Obac-induced RUNX2 expression in osteoblast differentiation (Figure 6C,D). Consistent with the findings, immunofluorescence observation also demonstrated that Dkk-1 and noggin attenuated the nuclear localization of RUNX2 in response to the treatment of Obac in osteoblast differentiation (Figure 6E, Supplementary Figure S3).

## 3. Discussion

Mesenchymal stem cells (MSCs) and pluripotent cells can be differentiated into Osteoblasts [24]. Osteogenic growth factors, such as BMPs and Wnts, induce complex signaling pathways, and initiate differentiation to osteoblast lineages and bone formation [11,25,26,27,28]. BMP2-mediated signaling induces the phosphorylation of Smad1/5/8, the nuclear translocation of Smad1/5/8 and Smad4 complexes, and then the regulation of gene expression [29]. Wnts/β-catenin signaling induces the stabilization of cytoplasmic β-catenin by inhibiting GSK3β, and then β-catenin is translocated into the nucleus to regulate gene transcription [30]. Both BMP and Wnt/β-catenin signaling have been linked to RUNX2 transcription factor in osteoblast differentiation and function [31,32]. BMP2 and β-catenin pathways upregulate the expression of RUNX2 gene, and also directly increase the transcriptional activity of RUNX2 [31,32,33]. RUNX2 is gradually expressed from MSCs to pre-osteoblasts and immature osteoblasts, and then RUNX2 is decreased in mature osteoblasts [34,35]. RUNX2 also induces cell-cycle arrest by the upregulation of p27 and suppresses cell proliferation in osteoblast lineages and osteosarcoma cells [36,37]. In the present study, we first identified the efficacy of Obac in osteoblasts, and demonstrated that Obac upregulates the expression of RUNX2 through BMP2-smad1/5/8 and β-catenin pathways in osteoblast differentiation. However, it might be considered in a future study whether Obac binds to a receptor or stimulates the phosphorylation of smad1/5/8 and β-catenin directly. These results suggest that Obac has biological activities, by the expression and nuclear accumulation of RUNX2 through the BMP and Wnt/β-catenin pathways.

The cell migration and recruit of pre-osteoblasts into specific niches are essential for osteoblast differentiation and bone formation [11,27,28]. In the present study, we demonstrated that Obac increases cell migration in osteoblast differentiation without the cell proliferation of MC3T3-E1. Obac also increased the activities and expression of ALP, which is known as a marker of the early osteoblast differentiation, and also increased matrix mineralization by the deposition of calcium phosphate mineral in the bone matrix as the phenotype of mature osteoblasts, which is a marker of the late osteoblast differentiation [15,38,39]. In addition, we demonstrated that the effects of Obac on osteoblast differentiation and RUNX2 are attenuated by Wnt/β-catenin inhibitor, Dkk-1 and a BMP inhibitor, Noggin. It was reported that RUNX2 acts as a transcriptional factor to regulate several osteoblast marker genes for osteoblast differentiation such as ALP, BSP, collagen I, OCN, OPN [33,40,41]. RUNX2 knock-out mice impaired bone ossification with the reduction in OCN [42]. We also found that Obac induced nuclear accumulation of RUNX2 and its target genes including ALP, BSP, OCN; OPN increased in osteoblast differentiation. These results suggest that Obac promotes osteoblast migration, differentiation, and function by upregulation of RUNX2 through the BMP and Wnt/β-catenin pathways to control bone repair, remodeling, and formation.

In conclusion, we originally demonstrated that Obac from the dried root bark of *D. dasycarpus* has biological activities including migration, differentiation, gene expression, and mineralization to stimulate osteoblast differentiation by the upregulation of RUNX2 in MC3T3-E1. Osteoblasts actively participate in the bone formation and repair by complex events: the migration and proliferation of pre-osteoblasts, osteoblast differentiation, and then the synthesis, secretion, and mineralization of the bone matrix [10]. Dysregulation in complex processes causes pathogenesis in bone diseases such as osteoporosis and periodontal disease [43,44,45,46]. Thus, our findings suggest that Obac might be a phytotherapeutic compound for drug development based on biological mechanisms to treat bone diseases such as osteoporosis and periodontal disease.

## 4. Materials and Methods

### 4.1. General Material for Extraction and Isolation from Dictamnus dasycarpus Turcz

The organic solvents, including methanol (MeOH), hexane (Hx), ethyl acetate (EtOAc), butanol (BuOH), and methylene chloride (MC) were purchased from Duksan Chemical Co. (Seoul, Korea). The column chromatography used was silica gel 60 (Merck 230–400 mesh, ASTM, Darmstadt, Germany). The preparative TLC was performed using 20 × 20 cm plates coated with 1 mm-thick F254 silica gel (Merck, Darmstadt, Germany). The NMR spectra were recorded on a JEOL ECX-500 spectrometer, operating at 500 MHz for ^1^H and 125 MHz for ^13^C NMR spectra (JEOL Ltd., Tokyo, Japan). The determination of high-performance liquid chromatography (HPLC) spectra were recorded on an Agilent 1260 series system (Agilent Inc., Palo Alto, CA, USA) with a photodiode array (PDA) and an evaporative light scattering detector (ELSD).

### 4.2. Extraction and Isolation of Compound

The dried root bark of *Dictamnus dasycarpus* Turcz. (1.1 kg) was extracted with 95% MeOH for 18 h (1000 mL). The MeOH extract (65 g) was suspended with 1000 mL of distilled water and solvent partitioned with the same volume of Hx, EtOAc and BuOH. The EtOAC soluble fraction (12 g) was separated into 9 fractions (DDE 1-9) by chromatography on a silica gel column eluted with a gradient of Hx and EtOAc (10:0 to 0:10, v/v). The DDE 3 was isolated by silica gel column eluted with isocratic solvent condition Hx and MC (10:1, v/v) to yield 7 fractions (DDE 3-1 ~ 3-7). The subfraction DDE 3-1 was purified by recrystallization in methanol to obtain obacunone (25 mg, purity: 99.8%).

### 4.3. Obacunone (Obac)

White powder; EI-MS *m*/*z* = 454.5 [M]^+^, molecular formula C_26_H_30_O_7_; ^1^H NMR (500 MHz, CDCl3) *δ* 7.43 (1H, t, H-21), 7.28 (1H, s, H-23), 6.53 (1H, d, *J* = 11.7 Hz, H-1), 6.36 (1H, s, H-22), 5.97 (1H, d, *J* = 11.0 Hz, H-2), 5.45 (1H, s, H-17), 3.66 (1H, s, H-15), 2.99 (1H, t, *J* = 11.0 Hz, H-6b), 2.60 (1H, dd, *J* = 4.0, 11.0 Hz, H-5), 2.30 (1H, dd, *J* = 4.0, 11.0 Hz, H-6a), 2.15 (1H, dd, *J* = 3.5, 8.5 Hz, H-9) 1.50 (6H, s, H-29, H-30), 1.45(3H, s, H-28), 1.24 (3H, s, H-19), 1.12 (3H, s, H-18); ^13^C-NMR (125 MHz, CDCl3) *δ* 207.4 (C-7), 167.0 (C-3), 166.8 (C-16), 156.8 (C-1), 143.3 (C-23), 140.9 (C-21), 122.8 (C-2), 120.0 (C-20), 109.7 (C-22), 84.0 (C-4), 78.0 (C-17), 65.0 (C-14), 57.2 (C-5), 53.4 (C-15), 53.1 (C-8), 49.1 (C-9), 43.0 (C-10), 39.8 (C-6), 37.3 (C-13), 32.6 (C-12), 31.9 (C-30), 26.7 (C-19), 21.0 (C-29), 19.4 (C-28), 16.9 (C-11), 16.4 (C-18).

### 4.4. Culture of Pre-Osteoblasts, and Osteoblast Differentiation

MC3T3-E1 pre-osteoblasts (CRL-2593) purchased from the American Type Culture Collection (ATCC) (Manassas, VA, USA) were kindly provided by the Bioevaluation Center (Korea Research Institute of Bioscience and Biotechnology, Daejeon, Korea), and cultured in α-minimum essential medium (α-MEM) (WELGEME, Inc., Seoul, Korea)) without L-ascorbic acid (Sigma-Aldrich, St. Louis, MO, USA) supplemented with 10% fetal bovine serum (FBS), penicillin (100 units/mL), and streptomycin (100 μg/mL) at 37 °C in a humidified atmosphere of 5% CO_2_ and 95% air. Osteoblast differentiation was induced using osteogenic supplement medium (OS) containing 50 μg/mL L-ascorbic acid (L-AA) and 10 mM β-glycerophosphate (β-GP). The medium was replaced every 2 days during the incubation period as previously described [47].

### 4.5. MTT Assay

Cell viability was performed using 3-[4,5-dimethylthiazol-2-yl]-2,5-diphenyltetrazolium bromide (MTT) solution to detect NADH-dependent dehydrogenase activity, as previously described [48]. Absorbance was measured at a wavelength of 540 nm using the Multiskan GO Microplate Spectrophotometer (Thermo Fisher Scientific, Waltham, MA, USA).

### 4.6. Cell Migration Assay

Cell migration was accessed using a wound healing assay as previously described [47]. Briefly, the cells were wounded with a 200 μL pipette tip and washed with 1 × PBS to remove cell debris. After that, the cells were treated with Obac for 24 h at 37 °C in a humidified atmosphere of 5% CO_2_ and 95% air. Cell images were observed to quantify cell migration rate using a light microscope. The migration assay was also performed with some modifications using a Boyden chamber as previously described [49]. Briefly, the Nuclepore filter was coated with matrigel and the Boyden chamber was assembled with conditioned medium in each lower chamber, matrigel-coated Nuclepore filter facing upward, and medium-containing cells in each upper chamber. After incubating the Boyden chamber for 4 h, the cells were fixed in 10% formalin, and stained by 0.5% crystal violet. The cells that traveled past the filter were counted as migration cells using a light microscope.

### 4.7. Alkaline Phosphatase (ALP) Activity Assay

Osteoblast differentiation was induced using OS containing 50 μg/mL L-AA and 10 mM β-GP with Obac (1 and 10 μM) for 7 days. The cell lysates were performed according to the manufacturer’s protocol using an alkaline phosphatase activity colorimetric assay kit (Biovision, Milpitas, CA) as previously described [47].

### 4.8. ALP Staining Assay

Osteoblast differentiation was induced using OS containing 50 μg/mL L-AA and 10 mM β-GP with Obac (1 and 10 μM) for 7 days. ALP staining assay was performed as previously described [47]. Cells were fixed in 10% formalin, rinsed with distilled water, and incubated at 37 °C for 1 h in substrate solution for ALP reaction (Takara Bio Inc., Japan). The level of ALP staining was observed using a scanner and colorimetric detector (ProteinSimple Inc., Santa Clara, CA, USA).

### 4.9. Alizarin Red S (ARS) Staining

Osteoblast differentiation was induced using OS containing 50 μg/mL L-AA and 10 mM β-GP with Obac (1 and 10 μM) for 14 days. ARS staining was performed as previously described [50]. Briefly, cells were fixed in 10% formalin, rinsed with distilled water, and stained with 2% Alizarin red S (pH 4.2) (Sigma-Aldrich) for 20 min. The level of ARS staining was observed using a scanner and colorimetric detector (ProteinSimple Inc., Santa Clara, CA, USA).

### 4.10. Reverse Transcription-Polymerase Chain Reaction (RT-PCR)

Total RNA was extracted using the RNAqueous kit, and cDNA was synthesized using the High-Capacity RNA-to-cDNA kit (Applied Biosystems, Foster City, CA, USA) as previously described [51]. Quantitative real-time PCR was performed using a 7500 Real-Time PCR System (Applied Biosystems, Foster City, CA, USA). The sequences of the primers were as follows: *Alp*, F:ACACCTTGACTGTGGTTACTG, R: CCATATAGGATGGCCGTGAAG; *Bsp*, F:TGTTTGTAGTGGGCTTCTTCTT, R:TCCATCTAGTCCCAGCTCATAG; *Opn*, F:GAGGTGATAGCTTGGCTTATGG, R:TCCTTAGACTCACCGCTCTT, *Ocn*, F:ACACCATGAGGACCATCTTTC, R:CGGAGTCTGTTCACTACCTTATT; β-actin, F:AATGTGGCTGAGGACTTTG, R: GGGACTTCCTGTAACCACTTATT.

### 4.11. Western Blot Analysis

Western blot analysis was performed as previously described [52]. Briefly, equal amounts of lysates (20 μg) were resolved by sodium dodecyl sulfate-polyacrylamide gel electrophoresis (SDS-PAGE) and transferred to a polyvinylidene fluoride (PVDF) membrane (Millipore, Bedford, MA, USA). After blocking with 1 × TBS containing 0.05% Tween 20 (TBST) and 5% skim milk for 1 h at room temperature, the membranes were incubated overnight at 4 °C with the primary antibodies, washed with 1 × TBST, and then incubated with horseradish peroxidase (HRP)-conjugated secondary antibodies (1:5000, Jackson ImmunoResearch, West Grove, PA, USA) for 1 h at room temperature. Immunoreactive proteins were detected using an enhanced chemiluminescence (ECL) kit (Millipore, Bedford, MA, USA) and the ProteinSimple detection system (ProteinSimple Inc., Santa Clara, CA, USA). Primary antibodies were as follows (Park et al., 2020): β-catenin (1:1000, #8480, Cell Signaling Technology Beverly, MA, USA), β-actin (1:1000, #sc-47778, Santa Cruz Biotechnology), BMP2 (1:1000 CSB-PA0, CUSABIO, Houston, TX, USA), p-GSK3β (1:1000, #9336, Cell Signaling Technology), RUNX2 (O1L7F; 1:1000, #12556S, Cell Signaling Technology), p-Smad1/5/8 (1:2000, #13820S, Cell Signaling Technology).

### 4.12. Immunofluorescence

Immunofluorescence was performed as previously described [53]. Cells were fixed, permeabilized, and blocked with 3% BSA diluted in 1 × PBS for 1 h. The cells were incubated with an anti-RUNX2 antibody (1:200, Cell Signaling Technology, Beverly, MA, USA) overnight at 4 °C. After washing three times in 1 × PBS, the cells were incubated with an Alexa-Fluor 568-conjugated secondary antibody (1:500, Invitrogen, Carlsbad, CA, USA) for 2 h at room temperature, stained with DAPI (Sigma-Aldrich, St. Louis, MO, USA), washed three times, mounted on glass slides, and observed using a confocal microscope (K1-Fluo Confocal Laser Scanning Microscope, Korea).

### 4.13. Statistical Analysis

The data were analyzed using Prism Version 5 program (GraphPad Software, Inc., San Diego, CA, USA). All numeric values are presented as the means ± S.E.M. The statistical significance was determined using a Student’s unpaired *t*-test. A value of *p* < 0.05 was considered to indicate statistical significance.

## Data Availability

Data is contained within the article or Supplementary Material.

## Supplementary Materials

The following are available online at https://www.mdpi.com/1422-0067/22/5/2483/s1, Figure S1: Effects of Obac on cytotoxicity and proliferation in pre-osteoblasts, Figure S2: Magnified image of Figure 5C, Figure S3: Magnified image of Figure 6E.

## Abbreviations

ALP	Alkaline phosphatase
ARS	Alizarin red S
β-GP	β-glycerophosphate
BMP	Bone morphogenetic protein
BSP	Bone sialoprotein
L-AA	L-ascorbic acid
MSCs	Mesenchymal stem cells
MTT	3-[4,5-dimethylthiazol-2-yl]-2,5-diphenyltetrazolium bromide
Obac	Obacunone
OCN	Osteocalcin
OPN	Osteopontin
OS	Osteogenic supplement medium
RUNX2	Runt-related transcription factor 2
