# Supplementary material for: Limonoid Triterpene, Obacunone Increases Runt-Related Transcription Factor 2 to Promote Osteoblast Differentiation and Function
Source: Int J Mol Sci. 2021 Mar 2;22(5):2483. doi: 10.3390/ijms22052483 (PMC7957678; doi:10.3390/ijms22052483)

Supplementary Figure 1. Effects of Obac on cytotoxicity and proliferation in pre-osteoblasts. Pre-osteoblasts were incubated with Obac at concentrations of 1, 10, 30, and 100 μM for 24 h, and cell viability was measured by BrdU incorporation assay. Data are representative of three independent experiments, and values are expressed in the mean ± S.E.M. *, $p < 0.05$ indicates statistically significant difference, compared with the control.

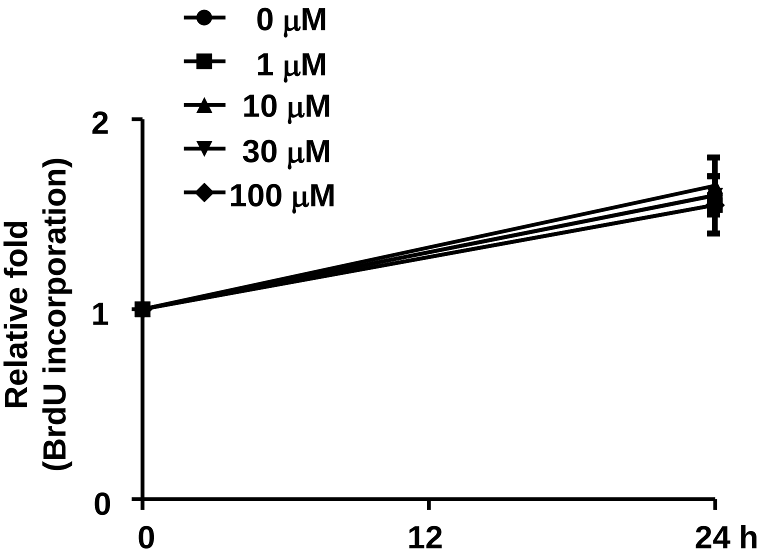

Supplementary Figure 2. Magnified image of Figure 5C

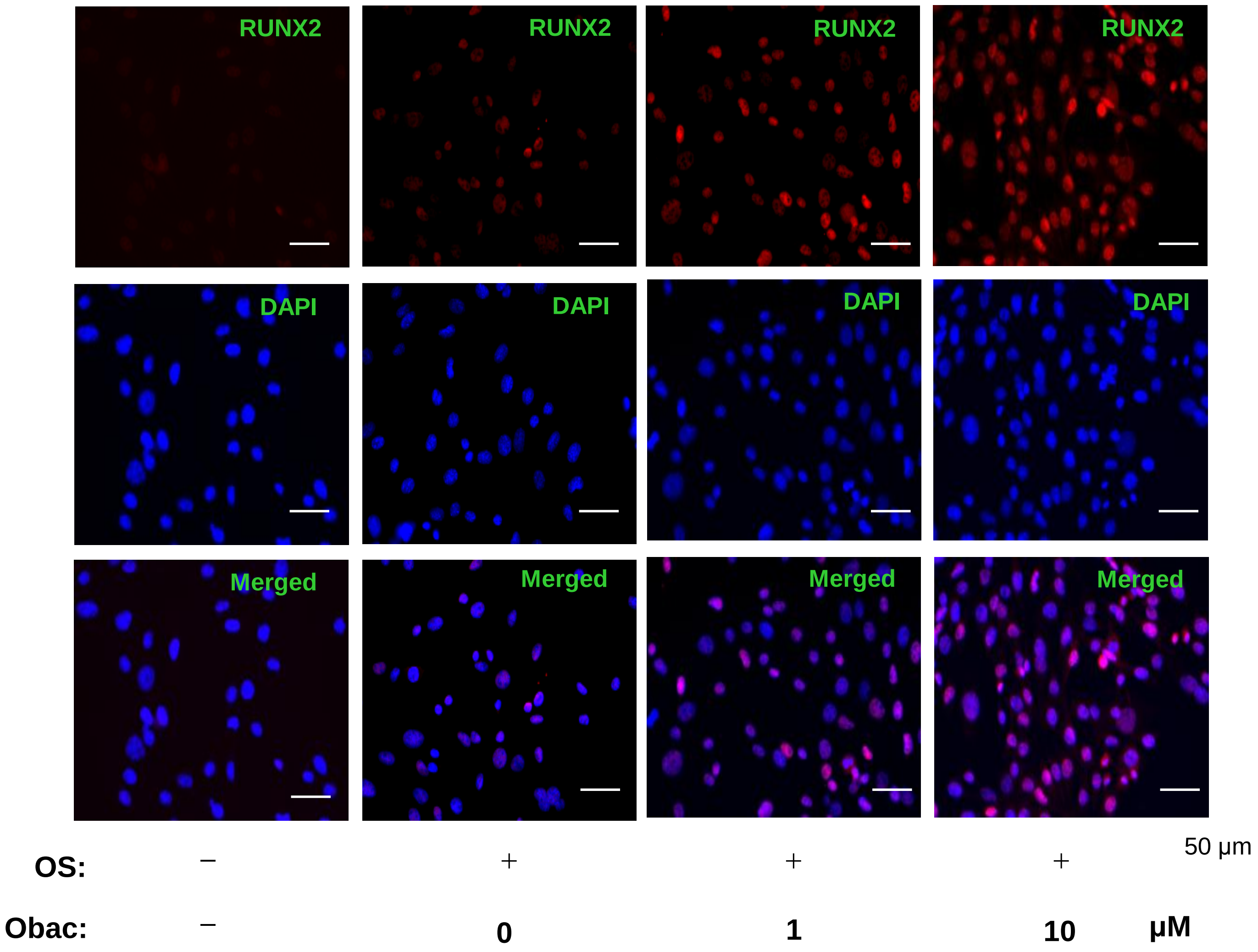

Supplementary Figure 3. Magnified image of Figure 6E

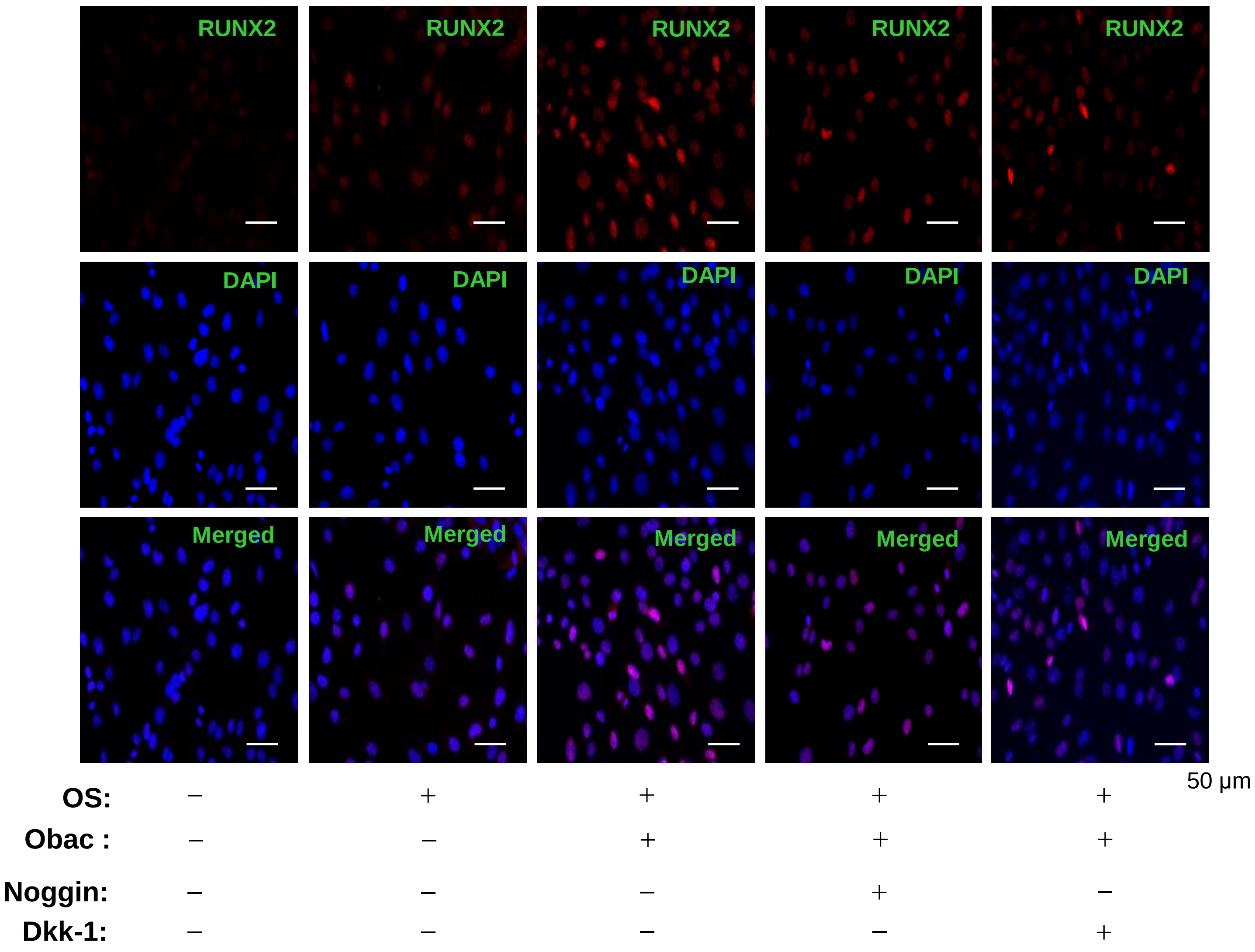

Supplement: Supplementary file 1 [file ijms-22-02483-s001.pdf]
